# Divergent Receiver Responses to Components of Multimodal Signals in Two Foot-Flagging Frog Species

**DOI:** 10.1371/journal.pone.0055367

**Published:** 2013-01-29

**Authors:** Doris Preininger, Markus Boeckle, Marc Sztatecsny, Walter Hödl

**Affiliations:** 1 Department of Integrative Zoology, University of Vienna, Vienna, Austria; 2 Department of Cognitive Biology, University of Vienna, Vienna, Austria; University of Sao Paulo, Brazil

## Abstract

Multimodal communication of acoustic and visual signals serves a vital role in the mating system of anuran amphibians. To understand signal evolution and function in multimodal signal design it is critical to test receiver responses to unimodal signal components versus multimodal composite signals. We investigated two anuran species displaying a conspicuous foot-flagging behavior in addition to or in combination with advertisement calls while announcing their signaling sites to conspecifics. To investigate the conspicuousness of the foot-flagging signals, we measured and compared spectral reflectance of foot webbings of *Micrixalus saxicola* and *Staurois parvus* using a spectrophotometer. We performed behavioral field experiments using a model frog including an extendable leg combined with acoustic playbacks to test receiver responses to acoustic, visual and combined audio-visual stimuli. Our results indicated that the foot webbings of *S. parvus* achieved a 13 times higher contrast against their visual background than feet of *M. saxicola*. The main response to all experimental stimuli in *S. parvus* was foot flagging, whereas *M. saxicola* responded primarily with calls but never foot flagged. Together these across-species differences suggest that in *S. parvus* foot-flagging behavior is applied as a salient and frequently used communicative signal during agonistic behavior, whereas we propose it constitutes an evolutionary nascent state in ritualization of the current fighting behavior in *M. saxicola*.

## Introduction

In order to understand the evolution of multimodal signals it is fundamental to investigate receiver responses to individual signal components. The individual components and their interactions with one another can have varying effects on receivers [1], [2], [3]. Three primary hypotheses have been suggested for explaining the evolution of multimodal signals and providing a signal classification framework: the content-based hypothesis, the efficacy-based hypothesis and the inter-signal interaction hypothesis [4], [5], [6], [7], [8]. The content-based hypothesis relates to the message of signal components and the response they elicit in receivers and classifies the function of signal components as “redundant” (“back-up”) or “non-redundant” (“multiple”) messages. The efficacy-based hypothesis addresses signal efficacy related to the environment, e.g. signals can either solve different transmission problems or act as a backup in varying environmental conditions. The inter-signal interaction hypothesis assumes that the signals do not always act independently, but the presence of one signal component alters the receiver's response to the second component, for example, increases detection and discrimination.

Recent studies on multimodal signaling have focused on the role of signals in female mate choice decisions. In particular behavioral experiments in wolf spiders using visual and seismic signals during courtship have provided profound insights into the evolution and function of multimodal signaling across species [1], [3], [9], [10]. Very little is known about multimodal signaling in male-male competition and agonistic interaction; especially how isolated signal components influence receivers remains poorly understood. Male territoriality or spacing behaviors often involve long distance signals [11], that are less suitable to experimental manipulation than signals involved in close range mate attraction. Another problem in understanding receiver response to multimodal signals comes from the fact that similar signal components have differing functions across species [2], [3]. Comparing responses to multimodal signal components across species may therefore allow more general conclusions about signal function and evolution to be drawn.

Anuran amphibians are excellent model systems to study multimodal communication, since all anuran species performing visual displays also use acoustic signals [12]. In particular the vocal sac has been shown to simultaneously serve acoustic as well as visual roles in mate attraction or territoriality [13], [14], [15], [16]. The linkage of acoustic and visual signal modes to the same organ makes it difficult to study the two channels. However, experimental studies on multimodal signals in *Allobates femoralis* have successfully disentangled receiver responses to the two signal components [13], [17]. Some frog species perform visual displays with their feet, independently from sound production known as foot flagging [12], [18], [19], [20]. How the isolated visual signal component influences male agonistic behavior has not been studied, but it was suggested that the call alerts the receiver to the subsequent foot-flagging signal in the genus *Staurois* [19], [20], [21]. Foot-flagging displays have been reported from 16 anuran species [12], [20], [22], [23], [24]. The behavior probably evolved convergently in five anuran families mostly inhabiting fast-flowing streams [12]. The Bornean Rock Frog (Ranidae: *Staurois parvus*) and the Small Torrent Frog (Micrixalidae: *Micrixalus saxicola*) from the Western Ghats of India belong to different anuran families. Males of both species use a complex signaling repertoire consisting of high pitched calls, foot flagging, and tapping (foot lifting) to signal the readiness to defend perching sites against other males [21], [22], [25]. Acoustic communication in the two species is not impaired by ambient low-frequency dominated stream noise, but concurrently chorusing conspecifics are suggested to constrain vocal communication in *M. saxicola* [25]. The conspicuously white colored foot webbings of *S. parvus* present a strong contrast to the dark body coloration whereas the feet of *M. saxicola* do not differ from the general body coloration as judged by the human eye. Previous studies have demonstrated that both species respond to acoustic playbacks, however, *M. saxicola* only displayed foot-flags if the acoustic signal was accompanied by a visual cue of a pulsating vocal sac [25]. Additionally, males of *M. saxicola* repeatedly attack each other with leg kicks (Preininger unpublished data) a behavior that has not been observed in *S. parvus*.

The aim of our study was to test how isolated unimodal signal components and their multimodal interactions influence male response in *M. saxicola* and *S. parvus*. Since visual signals may not be obvious to the human eye, for instance due to our lack of sensitivity to UV light, we first measured spectral reflectance of foot webbings and the visual background in both species using a spectrophotometer. We then performed behavioral field experiments for which we employed a model frog with an extendable leg combined with acoustic playbacks to present acoustic, visual and audio-visual multimodal stimuli to the frogs. As the tapping behavior was too complex to be performed by the experimental set-up, we restricted the visual stimulus to foot flagging. Attaching a white or a dark grey foot to the model's leg enabled us to manipulate the visual signal's conspicuousness and to explore the role of signal efficacy in receiver response. By comparatively describing the visual signal components as well as the response behavior, we discuss across-species differences and hypothesize that foot flagging in *M. saxicola* presents a nascent state in evolution of multimodal signaling.

## Methods

### Ethics statement

The behavioral experiments were performed without physical contact with the study animals. The experimental protocol adhered to the Animal Behaviour Society guidelines for the use of animals in research and all necessary permits were obtained for the described field studies and approved by the Universiti Brunei Darussalam Research Committee, the authority responsible for the Ulu Temburong National Park (permission number: UBD/PNC2/2/RG/1(58)) and the Centre for Ecological Sciences, Indian Institute of Science, Bangalore and Principal Chief Conservator of Forest (Wildlife), Karnataka State Forest Department, Government of Karnataka, the relevant regulatory bodies concerned with protection of wildlife for the Kathalekan swamp forest (permission number: D.WL.CR-27/2008-09).

### Study sites and species

#### *Staurois parvus*

The Bornean Rock Frog is a ranid frog, endemic to Borneo, recently resurrected from synonymy with *S. tuberilinguis* [26], [27]. We studied a population of *S. parvus* from March – April 2010 in the Ulu Temburong National Park, Brunei Darussalam, Borneo. The study site was situated at a narrow, rocky (black shale) section of the Sungai Mata Ikan, a small freshwater stream that merges into the Belalong River close to the Kuala Belalong Field Studies Centre (115°09′ E, 4°33′ N). The snout-urostyle length (SUL) and body mass of the investigated population of male *S. parvus* averaged 21.5 mm (SD ±0.5, n = 13) and 0.7 g (SD ±0.05, n = 13) respectively [21]. Males are diurnal and perch on rocks along fast-flowing forest streams. Their white chest and white webbintg between toes of the hind legs strongly contrast to their cryptic dark grey, brown dorsal body. Foot-flagging signals are mainly displayed during male-male agonistic interactions. We measured inter-individual distance between visually signaling males to determine average receiver distance. Median distance between advertising individuals in the study population was 0.93 m (range: 0.17–3.44 m, n = 11). The acoustic and visual displays are functionally linked in the genus *Staurois* as the call is suggested to alert the receiver to the subsequent foot-flagging signal [19], [20], [21].

#### *Micrixalus saxicola*

The second study species belongs to the family Micrixalidae and is endemic to the Western Ghats in India [28]. A population of the Small Torrent Frog was investigated at the end of the monsoon season (September – October 2010). *Micrixalus saxicola* occurs exclusively along small, fast-flowing streams within evergreen forests [29]. Individuals are diurnal and inhabit perennial streams characterized by low water, air and soil temperature in which they produce advertisement calls from exposed sites on rocks. Besides foot flagging males also kick other males in agonistic interactions [25]. Our study population was located at the Kathalekan *Myristica* swamp forest (14.27414° N, 74.74704° E) in the central Western Ghats, which is considered a relict forest. Males of the study population have an average SUL of 23.6 mm (SD ±0.6, n = 13) and a mean mass of 1.1 g (SD ±0.14, n = 13) and display a bright white vocal sac during vocalization. Inter-individual distance between calling males and males responding with foot-flagging signals was measured to determine average receiver distance. Median distance between advertising individuals in the study population was 0.19 m (range: 0.07–0.68 m, n = 15).

### Spectral reflectance measurements

We captured 13 *S. parvus* and 13 *M. saxicola* during nightly censuses while they were resting on leaves or rocks along the stream banks and kept them in terraria until the next morning. Catching the very agile and shy frogs in streams and waterfalls is almost impossible during the day while they are active. To avoid possible color changes occurring at night, we measured spectral reflectance using an Ocean Optics Jaz spectrometer (Ocean Optics, Dunedin, FL, USA) during daytime in the lab. The spectrometer had an integrated pulsed xenon light source (Jaz-PX) with a spectral response from 190–1100 nm. The reflectance data were collected from 300–700 nm and expressed in per cent relative to a white standard (WS-1 Diffuse Reflectance Standard, Ocean Optics). We used a custom made shield placing the reflection probe constantly at a 45° angle and in 5 mm distance to the frog's skin surface in order to reduce specular reflectance. The shield completely touched the frog skin preventing stray light from entering. To measure the coloration of each frog, we used the mean of three spectral reflectance scans for each of two body parts: the dorsal skin on the frog's back as a proxy of the frog's general body coloration and the mean of the foot webbings of both feet. Additionally, we took 10 reflectance measurements of dry spots on the pebbles and rocks from which the frogs signaled to describe the visual background. To measure how a visual signal is perceived by an animal, knowledge of the ambient light (i.e. the irradiance), the background against which the signal is presented, and cone sensitivities is required. As the spectral sensitivity of our study species' retina is unknown we did not use sophisticated visual models. Instead we calculated brightness contrasts between the frogs' foot webbings and backs and the rocks on which they signaled by taking the difference between the mean reflectance spectrum of a frog's body part and mean background reflectance divided by the sum of the same two quantities [30]. The brightness or the intensity of the reflectance spectrum (calculated as the area under the spectral curve) accounts for all the light being reflected from a surface. The calculated contrast index ranges from 1 to −1 and indicates if a body part is lighter (positive values) or darker than the background (negative values). Similarly, we calculated the contrast achieved by the model frog feet to their background (the loudspeaker housing).

### Model frog experiments

#### Experimental design

**Figure 1:**
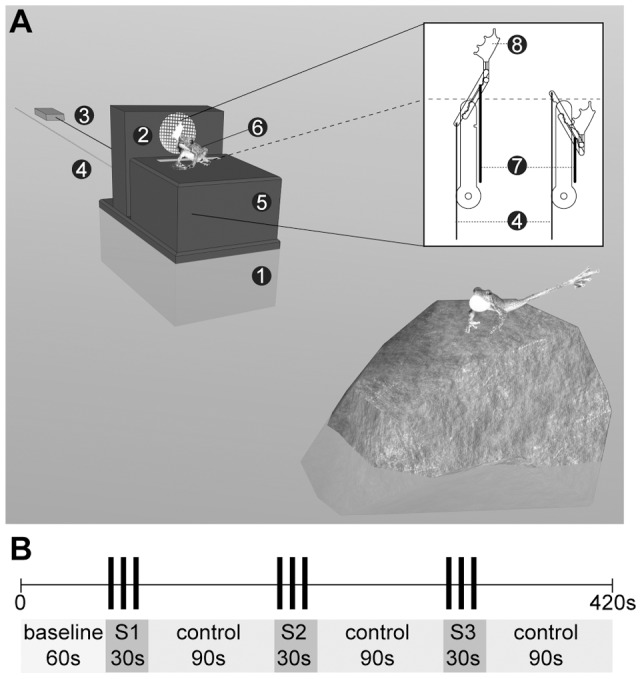
Schematic of the experimental set up and stimulus presentation. (A) The set up was positioned 50 cm from the focal individual. In the stream the lower box (1) serves as anchor for the upper set-up and a loudspeaker (2) connected to an portable player (3). A string (4) operated by the experimenter inserted through the upper box (5) stretched the artificial leg behind a model frog (6). A rubber band (7) automatically pulled back the leg and the attached foot (8). (B) After a 60 s baseline of no response the stimuli (S; acoustic, visual and multimodal) were presented for 30 s followed by a 90 s control period. Stimuli conditions were counterbalanced between positions S1, S2 and S3.

The experimental set up (Fig. 1A) consisted of two containers that formed a platform holding a model frog, an extendable artificial leg and a loudspeaker. The larger container (7×18×11 cm) was filled with pebbles and placed in the stream where it served as an anchor for the attached smaller container (6 cm ×10 cm ×11 cm) and the loudspeaker (Sony SRS-M 30) connected to a portable player (Odys Pax). On the smaller container we placed a stationary model frog as additional visual stimulus. To make the model frog, we created a silicone cast from a preserved specimen of *S. parvus* and filled it with Polyurethan resin (Neukadur MultiCast 1, Altropol, Stocklsdorf, Germany). Since *S. parvus* and *M. saxicola* males have similar body size, we used identical models for all experiments but painted them with acrylics according to previously taken photographs from the respective species. Finally, a clear coat was sprayed over the models to protect the paint from water and add a realistic sheen. Under the smaller container an extendable artificial leg made of sheet metal (0.25 mm thick) was affixed. The upper part of the leg including the exchangeable foot could be extended via a string by the experimenter and was pulled back automatically by a rubber band (Fig. 1A enlarged image).

#### Experiments and play-back stimuli

The experimental set-up was placed 50 (±5) cm from a focal male individual in the stream (sender-receiver distance in the range of the study species) and the experimenter operated play-backs from a distance of 1.5 m. Experimental presentations started when the focal individual showed no signaling behavior for a period of 60 s. We presented each individual with three stimuli: two unimodal stimuli (acoustic/visual) consisting in each case of either three calls or three foot flags and one multimodal stimulus (combined acoustic and visual) consisting of three composite (call and foot flag) presentations. Each stimulus presentation lasted 30 s followed by a 90 s control period (Fig. 1B) followed by the next stimulus adding up to a total duration of 420 s (incl. 60 s baseline) for one experiment. To control the experimental design for order effects in repeated measurements, the stimuli were presented in differing order to the tested individuals. The three advertisement calls for the acoustic stimulus had an intensity of 75 dB at 50 cm for *M. saxicola* and 70 dB at 50 cm for *S. parvus* corresponding to the average call intensity of the study population at a distance of 50 cm. We played back wav files of noise-reduced, pre-recorded advertisement calls and selected calls with average call characteristics from a greater sample (*M. saxicola* (n = 6): call duration: 2.6 s, note number: 21, mean dominant frequency: 4.6 kHz, intercall interval 7.4 s; *S. parvus* (n = 4): call duration: 6.1 s, note number: 35, mean dominant frequency: 5.5 kHz, intercall interval 3.9 s). Each foot-flag lasted 2 s (time between raising and retracting the artificial leg; inter-signal interval 8 s). For the combined multimodal stimulus we presented an advertisement call immediately followed by a foot-flag (inter-signal interval 2 s between multimodal stimuli). Average call parameters and foot-flagging durations were representative of our two study populations [21], [25].

**Figure 2:**
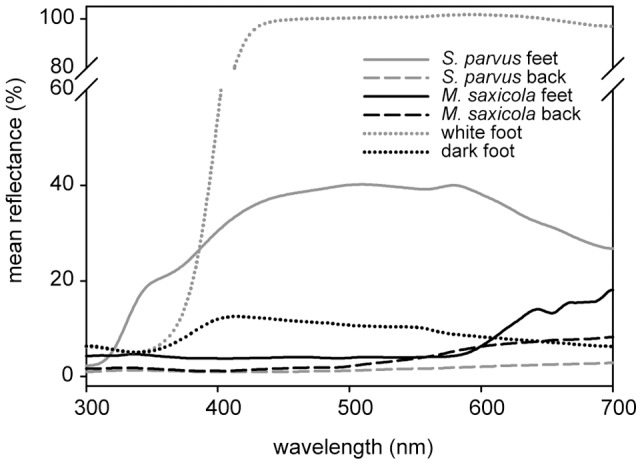
Reflectance spectra of the white (**grey dotted line**) **and dark model foot** (**black dotted line**) **used in the experimental playback presentations;** ***Staurois parvus*** **feet** (**grey solid line**) **and back** (**grey dashed line**)**;** ***Micrixalus saxicola*** **feet** (**black solid line**) **and back** (**black dashed line**)**.** N = 13 in both species.

To test if the brightness of interdigital webbings has an influence on response frequencies, we conducted experimental presentations with individuals of both species using a white (100% reflection of light from 465–650 nm compared to the white standard) and a dark-grey (ca. 10% light reflection) artificial foot during visual and multimodal stimuli (Fig. 2). Commercial paints absorb in the UV and we added Barium sulfate (ReagentPlus, 99%, Sigma Aldrich, Germany) to our acrylics to boost the UV component and achieve a more even reflection between 300 (UV) and 700 nm (red). As BaSO_4_ increases reflection in all wavelengths, we could only add so much as to adjust the overall brightness to 100% and 10% compared to our white reflectance standard respectively.

#### Data collection and analysis

All trials were video recorded with a waterproof camera (Sanyo Xacti WH1) positioned on a tripod. Dorsal patterns of frogs allowed individual recognition in order to avoid multiple testing of the same individual. We analyzed frequencies of the behavior categories “calling”, “tapping”, “foot-flagging” during stimulus and control periods with the behavioral coding software Solomon Coder [31]. “Tapping” constitutes the lifting of either the right or left leg without stretching it, whereas “foot-flagging” describes the behavior of completely extending the leg above and back in an arc and bringing it back to the body side [12]. For statistical analysis we only used data from recordings in which the focal individual could be observed for the complete experimental presentation. We analyzed responses of 16 *M. saxicola* males; 8 playback presentations were conducted with the white foot and 8 with the dark-grey foot. In *S. parvus* 31 males were tested, and 14 experiments were performed with the white foot and 17 with a dark-grey foot.

To test whether the frequency of responses is dependent on species (*M. saxicola* and *S. parvus*), brightness of foot (white and dark) and/or stimulus (acoustic, visual and multimodal), we calculated zero inflated Generalized Linear Mixed Models (GLMMs) with a poisson distribution and a log link function. We used the glmmADMB package [32] within the R statistical software [33]. The glmmADMB package allows for the simultaneous modeling of random effects and the overabundance of zeros in count data (i.e. zero inflation). The response variables “call”, “tap”, “foot flag” and the sum of responses (“call”, “tap” and “foot flag”) were modeled in four model sets for dependence on predictor variables using a backward step-wise selection procedure. The global model consisted of all predictor variables (species, brightness and stimuli) and their two-way interactions (Table 1). We started with the global model and excluded each predictor with a significance value *P*>0.1. In case we encountered significant interactions between the predictor variables we split the data accordingly into subsets in order to calculate significant effects within the subset. Terms were only regarded as being significant if *P*<0.05. To correct for the differences between individuals we included the nested term species (individual) as random variable for all models with the exception of models performed for the response variable foot-flag. *Micrixalus saxicola* displayed no foot-flagging behavior during playback presentation and only responses of the subset *S. parvus* were corrected with the random effect (individual). From the log likelihood of each model we calculated the small sample Akaike's Information Criterion (AIC_c_) to rank the models (the model with the lowest AIC_c_ value is the best supported by the data [34]). The absolute value of AIC_c_ is not relevant; it is the difference in AIC_c_ between models *i* and the model with the lowest AIC_c_ value (AIC_cmin_) (ΔAIC_c*i*_  =  AIC_c*i*_ – AIC_c*min*_) that gives information whether a model is relatively well or poorly supported by the data. Models with ΔAIC_c_ ≤2 can be considered to have substantial support for interpretation [34]. We also calculated Akaike weights (ω*_i_*) that are data-dependent, posterior model probabilities used to calculate evidence ratios ω*_i_*/ω*_j_* (a ratio of 3/1 would suggest that one model is three times better supported by the data than the other model; [34]). In the best model, predictors and interactions remained regardless of their significance and the results of pair-wise comparisons of this final model are presented (Table 2).

**Table 1:** Backward step-wise model selections obtained from Generalized Lineal Mixed Models to explain the frequency of single response behaviors (call, tap, foot flag) and their sum as function of species (*Micrixalus saxicola*, *Staurois parvus*), artificial foot brightness (dark, white), stimuli (acoustic, visual, multimodal) and their interactions.

Variable	Random Factor	Subset	Model	AICc	ΔAICc	ω
Sum	(Species(Individual))		Species + Brightness + Stimuli +Species:Brightness + Species:Stimuli + Brightness:Stimuli (Full model)	517.41	10.00	0.0033
			Species + Brightness + Stimuli + Species:Brightness + Species:Stimuli	513.24	5.83	0.0269
			Species + Brightness + Stimuli + Species:Brightness + Brightness:Stimuli	514.95	7.55	0.0114
			Species + Brightness + Stimuli + Species:Stimuli + Brightness:Stimuli	514.99	7.58	0.0112
			Species + Brightness + Stimuli + Species:Brightness	511.92	4.51	0.0520
			Species + Brightness + Stimuli + Species:Stimuli	510.89	3.48	0.0869
			Species + Brightness + Stimuli + Brightness:Stimuli	512.60	5.19	0.0370
			Species + Stimuli + Species:Stimuli	508.59	1.18	0.2752
			**Species + Stimuli**	**507.41**	**0**	**0.4961**
Call	(Species(Individual))		Species + Brightness + Stimuli + Species:Brightness + Species:Stimuli + Brightness:Stimuli (Full model)	323.59		
	(Species(Individual))	White	**Species + Stimuli + Species:Stimuli**	**147.78**	**−0.20**	**0.5248**
			Species + Stimuli	147.98	0.00	0.4752
		Dark	Species + Stimuli + Species:Stimuli	172.31	7.12	0.0217
			Species + Stimuli	167.70	2.50	0.2177
			**Species**	**165.20**	**0**	**0.7606**
Tap	(Species(Individual))		Species + Brightness + Stimuli + Species:Brightness + Species:Stimuli + Brightness:Stimuli (Full model)	175.22	10.37	0.0029
			Species + Brightness + Stimuli + Species:Brightness + Species:Stimuli	170.53	5.67	0.0300
			Species + Brightness + Stimuli + Species:Brightness + Brightness:Stimuli	171.68	6.83	0.0169
			Species + Brightness + Stimuli + Species:Stimuli + Brightness:Stimuli	172.96	8.11	0.0089
			Species + Brightness + Stimuli + Species:Brightness	168.09	3.23	0.1017
			Species + Brightness + Stimuli + Species:Stimuli	168.16	3.31	0.0979
			Species + Brightness + Stimuli + Brightness:Stimuli	169.61	4.76	0.0474
			Species + Brightness + Stimuli	216.73	51.88	0.0000
			Species + Stimuli + Species:Stimuli	167.46	2.60	0.1394
			Species + Brightness + Species:Brightness	-	-	-
			Brightness + Stimuli + Brightness:Stimuli	169.81	4.96	0.0429
			**Species + Stimuli**	**164.85**	**0**	**0.5121**
			Brightness + Stimuli	211.60	46.75	0.000
			Stimuli	215.77	50.91	0.000
Foot flag	Individual	*S. parvus*	Brightness + Stimuli + Brightness:Stimuli (Full model)	245.08	4.84	0.0631
			Brightness + Stimuli	242.53	0.29	0.2266
			**Stimuli**	**240.24**	**0**	**0.7103**

AIC_c_ based model rankings are shown. Predictor variables of the model with the lowest Akaike weight (ω) support best the frequency of response behaviors. The final models are presented in bold.

**Table 2:** Pair-wise comparisons of predictors and interactions of final models based on stepwise model selections (see Table 1).

Variable	Subset	Coefficients (Reference level)	Estimate	SE	z-Value	P-Value
Sum		Intercept	1.653	0.209	7.92	2.3e-15 ***
		Species *S.p.* (*M.s.*)	−0.840	0.257	−3.27	0.00108 **
		Stimulus A (M)	0.645	0.160	4.04	5.3e-05 ***
		Stimulus V (A)	−0.640	0.177	−3.61	0.00031 ***
		Stimulus M (V)	−0.006	0.197	−0.03	0.9777
Call	White	Intercept	1.418	0.226	6.27	3.7e-10 ***
		Species *S.p.* (*M.s.*)	−1.521	0.485	−3.14	0.0017 **
		Stimulus A (M)	0.332	0.317	1.05	0.2953
		Stimulus V (A)	−0.727	0.533	−1.36	0.1731
		Stimulus M (V)	0.395	0.533	0.74	0.4583
		Species *S.p.* (*M.s.*): Stimulus A (M)	1.042	0.899	1.16	0.2462
		Species *S.p.* (*M.s.*): Stimulus V (A)	−12.326	238.290	−0.05	0.9587
		Species *S.p.* (*M.s.*): Stimulus M (V)	11.284	238.290	0.05	0.9622
	Dark	Intercept	0.762	0.465	1.64	0.1
		Species *S.p.* (*M.s.*)	−3.045	0.731	−4.17	3.1e-05 ***
Tap		Intercept	0.953	0.858	1.11	0.267
		Species *S.p.* (*M.s.*)	−1.334	0.827	−1.61	0.107
		Stimulus A (M)	1.019	0.598	1.71	0.088
		Stimulus V (A)	−0.951	0.476	−2.00	0.046 *
		Stimulus M (V)	−0.069	0.697	−0.10	0.922
Foot flag	*S. parvus*	Intercept	0.788	0.178	4.43	9.3e-06 ***
		Stimulus A (M)	0.583	0.302	1.93	0.0540
		Stimulus V (A)	−0.859	0.323	−2.66	0.0079 **
		Stimulus M (V)	0.277	0.360	0.77	0.442

Estimates are given relative to the intercept. Significant differences between species (*Micrixalus saxicola* (*M.s*.), *Staurois parvus* (*S.p*)) and/or stimuli (acoustic (A), visual (V) and multimodal (M)) in the frequency of single behavioral responses (call, tap, foot flag) or their sum are marked with asterisks.

## Results

**Figure 3:**
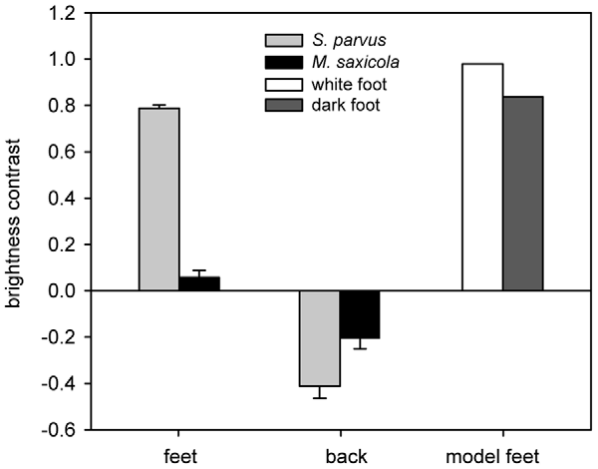
Mean brightness contrast of feet and back skin of *M. saxicola* and *S. parvus* against their natural visual background (**pebbles and rocks**) **and brightness contrast of the artificial model feet against the background of the experimental set-up** (**loudspeaker housing**)**.**

Foot webbings of *S. parvus* achieved a 13 times higher contrast against their visual background than feet of *M. saxicola* with differences between the species being highly significant (Fig. 3, t-test: *t* = −22.0, d.f. = 24, *P*<0.001). The backs of both species were darker than the visual background with the contrast being significantly larger in *S. parvus* than in *M. saxicola* (t-test: *t* = −3.02, d.f. = 24, *P* = 0.006).

During all playback experiments, the 16 tested *M. saxicola* males responded by performing a total of 125 calls (79%), 34 taps (21%) but no foot flags. The 31 *S. parvus* males tested displayed 21 calls, 19 taps and 83 foot flags, thus a playback was predominantly responded to with foot-flagging signals (68%) rather than calls (17%) or taps (15%). Four individuals of *S. parvus* responded five times with combined displays which could be regarded as multimodal signal (call and simultaneously performed foot flag). Due to the low occurrence of multimodal responses they were not analyzed separately but included to the respective response category call or foot flag.

**Figure 4:**
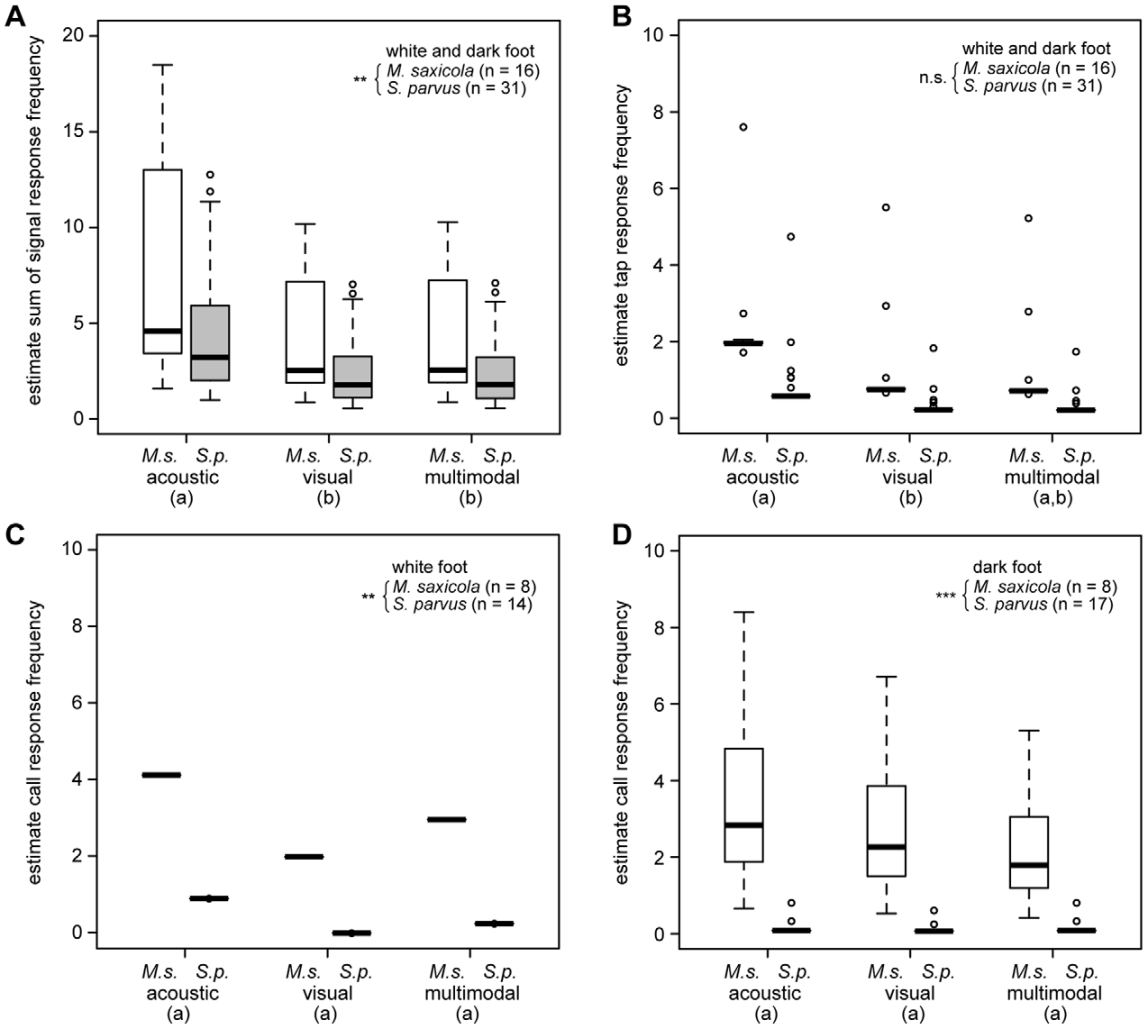
Comparison of response frequency of signal behaviors of *Micrixalus saxicola* (***M.s***) **and** ***Staurois parvus*** (***S.p***) **between acoustic, visual or multimodal stimuli.** (A) sum (call, tap and foot flag) response of white and dark foot playback presentations; (B) tap response of white and dark foot playback presentations; call response of (C) white foot and (D) dark foot playback presentations. Box plots show the estimated mean individual value with interquartile range, 10th and 90th percentile and minimum and maximum values, o designate outliners. Statistical significant response frequency differences between species are denoted by asterisk (** *P*<0.01; *** *P*<0.001), between stimuli the values without the same superscript letter (a, b) differ significantly at (A) *P*<0.001 and (B) *P*<0.05 (also see Table 2).

The frequency of the sum of behavioral responses was smaller in *S. parvus* compared to *M. saxicola* (GLMM: pair-wise comparison: *ß* = −0.840, SE = 0.257; *z* = 7.92, *P* = 0.001), and both species displayed more overall responses to acoustic stimuli than to visual stimuli (GLMM: pair-wise comparison: *ß* = 0.640, SE = 0.177; *z* = 3.61, *P*<0.001) and multimodal stimuli (GLMM: pair-wise comparison: *ß* = 0.645, SE = 0.160; *z* = 4.04, *P*<0.001; Fig. 4A). The frequency of tap responses did not differ between the tested species (Table 2). Both species responded with less tapping behavior to visual stimuli compared to acoustic stimuli (GLMM: pair-wise comparison: *ß* = −0.951, SE = 0.476; *z* = −2.00, *P* = 0.046) and the acoustic stimuli were answered with similar frequency as the multimodal stimuli (GLMM: pair-wise comparison: *ß* = 1.019, SE = 0.598; *z* = 1.71, *P* = 0.088; Fig. 4B).

In the global model calculated for call responses the predictors brightness and stimulus showed significant interactions and the model was split into the subsets white and dark. Response frequency between differing stimuli in the subsets showed no significant differences (Table 2).

**Figure 5:**
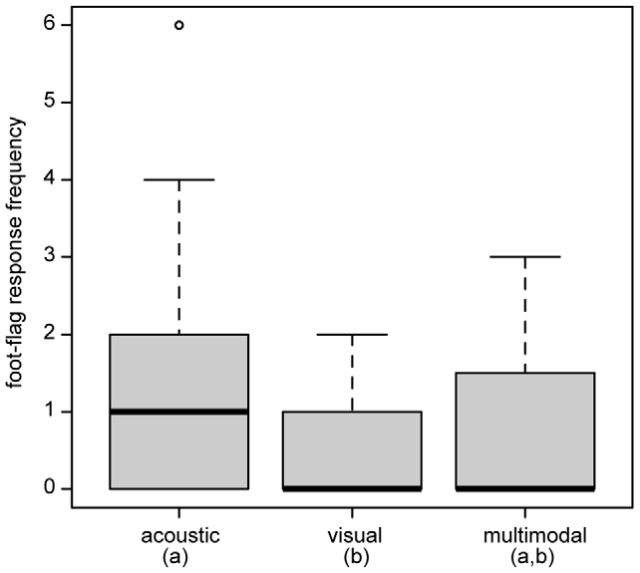
Comparison of foot-flagging response of *Staurois parvus* between acoustic, visual and multimodal stimuli. Box plots show the mean individual value with interquartile range, 10th and 90th percentile and minimum and maximum values. Values without the same superscript letter (a, b) differ significantly at *P*<0.01.

*Staurois parvus* performed fewer foot flags in response to visual stimuli compared with acoustic stimuli (GLMM: pair-wise comparison: *ß* = −0.859, SE = 0.323; *z* = −2.66, *P* = 0.007) and tended to respond more to acoustic stimuli than multimodal stimuli (GLMM: pair-wise comparison: *ß* = 0.583, SE = 0.302; *z* = 1.93, *P* = 0.054; Fig. 5).

## Discussion

*Micrixalus saxicola* and *S. parvus* are not closely related but share a similar breeding habitat and use similar, convergently evolved multimodal signals to communicate during male-male agonistic interactions. Despite similar ecological constrains, our study showed that a number of differences exist in visual signal conspicuousness and the response to identical signal stimuli between the tested species. The foot webbings of *S. parvus* contrasted much stronger against the background than those of *M. saxicola*. The main response to all experimental stimuli in *S. parvus* was foot flagging, whereas *M. saxicola* responded more actively to all tested stimuli conditions than *S. parvus* and responded primarily with calls (79% of all responses) but never foot flagged. The higher frequency of response in *M. saxicola* compared to *S. parvus* to the presented stimuli could result from seasonal differences in mating conditions in the habitat of the study species. Both species have observed to breed during and after heavy rains. Breeding season of *M. saxicola* coincides with monsoon rains (July–October) and population density increases during this period [22], [35]. Limited periods for mating during the rainy season and a basic necessity to secure signaling sites during that time where resources (e.g. shallow water areas or pools with gravel) are available for reproduction could have led to high levels of agonistic behavior towards the model as observed in this species. In northern Borneo, the habitat of *S. parvus*, dry periods are infrequent and short [36] and reproduction is not limited to a certain period of the year.

While across-species differences in our results are distinct, the minor within species differences in receiver behavior to the presented stimuli are not easy to interpret. Neither species responded to the stimuli with explicitly aggressive behavior such as attacking the model frog as found in the territorial dart poison frog *A. femoralis* [13], [17], or in studies on female mate choice in the túngara frog or wolf spiders [2], [3]. Instead our study species responded to all stimuli with a complex set of audio-visual signals. As response behavior can become more variable with increasing signal complexity [2], testing specific hypotheses related to multimodal signaling becomes a challenging task. Focusing on the primary response to stimulus types in the two investigated species, the call response frequency in *M. saxicola* did not differ between the three types of playback stimuli suggesting that the acoustic, visual and multimodal displays are of equal significance acting as redundant signal components. However, *S. parvus* displayed a higher number of foot flags during acoustic stimuli than visual presentations and signal frequency also tended to be less during multimodal stimuli. The primary use of foot flagging and the differences in response to the stimulus types indicate that acoustic and visual signals may be non-redundant. Previous studies on the genus *Staurois* suggested that the call acts as an alerting signal to the subsequent foot-flagging display which is supported by our findings [19], [20], [21]. Signal responses in both study species showed no *per se* qualitative difference (i.e. no response opposed to response) as observed in 11 taxa performing composite acoustic and visual signals (reviewed in [37]). Comparing our results to those on other species and drawing assumptions on signal message therefore is difficult and further experiments are needed.

The low levels of response to the multimodal stimulus and the lack of differences in response to the white and the grey model foot were unexpected results. Multimodal signals are assumed to elicit equal or enhanced responses in receivers compared to their unimodal components [7], [8], [38]. For instance female house crickets and wolf spiders were more attracted towards multimodal than unimodal male signals in mate choice experiments [3], [39]. During agonistic male-male interactions receiver responses to threat signals should depend on the distance between sender and receiver as well as the fighting technique of a species [40]. Aggressive kicking behavior in *M. saxicola* is only effective at close range to the opponent (lengths of the hind legs) and the distance between the model and actual frogs might have been too large to elicit aggressive response. The reduced frequency of foot-flagging in *S. parvus* to the visual and multimodal stimuli may indicate that the signals were not always perceived as a threat. No or reduced response does not necessarily suggest that the visual stimulus was not perceived (independently of the stimulus coloration) the experimental display could have been perceived as supernormal stimulus, hence as oversized opponent or the response may be graded. The low response could at least partly be a consequence of insufficient visual stimulus quality. The stimulus coloration did not exactly match the color of the frogs' feet. Furthermore the focal individuals perceived the model frog's foot mostly against the black loudspeaker and not against the natural background. As the loudspeaker housing was much darker than the surrounding pebbles and rocks the contrast achieved by both the grey and the white model foot was higher that that of the actual frogs' feet against the natural background (Fig. 3). This might explain why we found no difference in response frequency between the two experimental set-ups. Both artificial-model feet may have represented a supernormal visual stimulus. In addition, foot-flagging behavior of actual frogs is complex and the experimental set-up's leg movement was perhaps too simplified to elicit a natural response. Alternatively, the increased signal directionality and localizability of the visual stimulus [21], [25], may have caused the receiver to retreat rather than signal back [41], [42].

Signals used during aggressive or agonistic encounters reflect the species' fighting technique [43]. During the breeding season, males of *M. saxicola* occur in higher densities than *S. parvus* and engage in numerous close-range agonistic interactions with individuals performing acoustic and visual signals [22]. Male-male signaling is often preceded by physical attacks during which individuals kick opponents off the rocks with their hind legs (Preininger unpublished data). We therefore suggest that foot flagging may have evolved via ritualization from aggressive kicking behavior during male combat [12]. Ritualization is predicted to be the most common process for the evolution of animal signals [44] during which cues are thought to be modified to enhance their efficacy [43], [45]. Ritualized communication signals are expected to show increased conspicuousness, redundancy, and stereotypy compared to the original cue and additional alerting signal components may occur [11], [43]. A signal displayed during agonistic interactions should improve communication thereby reducing energy costs [46] and lead to lower rates of attacks and injury as shown in jumping spiders (*Phidippus clarus*) [47]. We never observed any kicking behavior in *S. parvus* and the foot-flagging signal was more salient than in *M. saxicola*, was displayed more frequently, and appears to be preceded by an alerting call [19], [20], [21]. Formal testing of the ritualization hypotheses requires a phylogenetic comparison across species with homologous behaviors [45], which is lacking in foot-flagging frogs. However, given the observed differences in our two study species we suggest that foot-flagging behavior in *M. saxicola* could constitute an evolutionary nascent state in ritualization of a communicative visual signal.

Male-male competition and agonistic interactions have rarely been considered a significant influence on multimodal signal evolution (but see [48], [49], [50], [51], [52]). In particular hypotheses on multimodal signal function were specifically set up in the context of courtship behavior and female mate choice [4], [5]. Testing content or efficacy based signal hypotheses in male aggressive signals in particular when receiver response involves complex signaling behavior might be more difficult than previously thought. The option to manipulate the distance between signaler and receiver in the study of aggressive signals appears important to draw conclusions on signal content. Signal characteristics could covary with physical parameters of the sender, as described for spectral and temporal properties of the advertisement call of several anuran species [53], visual signals in lizards [54], or vibratory signals in jumping spiders [50]. Future studies should investigate signal characteristics in relation to size and age of the signaler and explore signal function in regard to female responses. We believe that a comparative, across-species approach will help to explain multimodal signal evolution and will promote our understanding of how environmental selection pressures and sexual selection have influenced the evolution of the currently observed signal forms and functions.
